# A Compact L-Band Reconfigurable Dual-Mode Patch Filter

**DOI:** 10.3390/mi16111294

**Published:** 2025-11-19

**Authors:** Abdel Fattah Sheta, Majeed A. S. Alkanhal, Ibrahim Elshafiey

**Affiliations:** Electrical Engineering Department, King Saud University, Riyadh 11421, Saudi Arabia; majeed@ksu.edu.sa (M.A.S.A.); ishafiey@ksu.edu.sa (I.E.)

**Keywords:** reconfigurable filter, patch resonator, compact filter, dual-mode patch

## Abstract

This research presents a novel dual-mode filter design that offers significant advantages in terms of frequency agility and miniaturization compared to conventional fixed multi-resonator filters. The design and implementation of a compact tunable bandpass filter are presented. The basic design structure is based on a slotted non-degenerate dual-mode microstrip square patch. The slots are etched symmetrically, which makes the slotted dual-mode square patch equivalent to a two-coupled-resonator filter. The asymmetrical feed lines enable the excitation of dual resonant modes. The patch length, slot size, and dielectric material properties primarily determine the filter’s center frequency and bandwidth. Tunability is achieved by loading the slotted square patch with reversed bias varactor diodes located at the square patch corners, allowing electronic control of the filter center frequency. The design utilizes RT/Duroid 6010.2 laminates with a dielectric constant of 10.2 and a thickness of 0.635 mm. A bias tee at one of the filter ports is used to provide reverse bias to varactor diodes. Simulations and experimental results demonstrate tunable characteristics. Among the attractive features of the proposed design, good levels of insertion loss and impedance matching are noticed in the entire tunable band. The advantages of the proposed design make it well-suited for modern wireless technology applications in communication, radar, and satellite systems.

## 1. Introduction

The rapid growth of wireless technology systems has increased the demand for compact, tunable, and high-performance RF front-end components. Tunable bandpass filters (BPFs) play a dominant role in multi-carrier systems by selecting the desired band for a specific need. Conventional microstrip filters provide simplicity and integration advantages. However, they suffer from limited adaptability to changing frequency requirements. On the other hand, wideband filters, based on filter bank techniques, often require multiple resonators and switches, leading to increased size and design complexity. Reconfigurable filters have been proposed to address these limitations. Reconfigurable filters can dynamically adjust center frequency and bandwidth in response to system requirements. Various approaches have been investigated, including RF-micro-electro-mechanical system (MEMS) [1,2,3]. In addition, ferroelectric tunable materials have been proposed [4,5], as well as PIN diodes [6,7,8] and semiconductor varactors [9].

Varactor diode-based reconfigurable filters are the most appropriate technique for continuous control of the filter center frequency, low cost, and simple integration into microstrip technology. The continuous frequency control is achieved by varying the reverse bias voltage, and thereby varying the varactor capacitance [9]. Four-square ring resonators loaded with varactor diodes have recently been suggested for a tunable bandpass filter covering the range from 2.22 to 2.44 GHz [10]. The design relies on nine varactor diodes. Another fourth-order reconfigurable ring-coupled microstrip resonator is suggested in [11]. Tuning is performed by loading two rings with varactor diodes. A tuning range of 220 MHz is achieved. Reference [12] presents another varactor diode-based square ring resonator using a different arrangement. The filter offers a broad, tunable band suitable for operation below 3 GHz. The varactor-based filter tuning range is generally limited by the capacitance variation range of the varactor (varactor ratio). Three techniques, as mentioned in [13,14], can be applied to increase the tuning range, such as switchable resonators, zero value coupling, and resonator-based optimization. A switchable approach is used in [15,16,17] to offer a wide tuning range. PIN diodes are used as a switching device. Switchable and tunable quarter-wave resonators are utilized to design a wide-tunable bandpass filter in [18]. Switching is achieved by PIN diodes, while tuning is performed using varactor diodes. The technique allows a tuning range from 1.05 to 2 GHz. Dual-mode patch resonator filters have been proposed as compact, high-performance, narrow-band filters [19]. In this case, degenerate modes can be excited by various types of perturbations. A tunable dual-mode patch filter is also investigated based on degenerate modes as in [20], where varactor diodes achieve the tunability.

In this paper, a tunable microstrip patch bandpass filter is introduced. The filter is based on a nondegenerate dual-mode square microstrip patch with symmetrical slots [21,22]. Asymmetrical feed lines, which allow dual-mode operation by exciting multiple resonant frequencies, are used. Furthermore, varactor diodes are incorporated as a loading mechanism to provide continuous frequency tuning via capacitance control. This integrated configuration enables the filter to achieve tunability and reduced component count compared with conventional fixed or cascaded filter designs. The proposed design exhibits flexibility suitable for a wide range of RF and microwave applications, including satellite communications, wireless base stations, radar systems, and cognitive radio platforms. In addition, the adoption of dual-mode operation ensures that the filter achieves compactness and functional equivalence to higher-order multi-resonator filters.

## 2. Dual-Mode Microstrip Patch Resonator

Microstrip patch resonators have been proposed for compact and low-cost bandpass filter applications. Various shapes of resonators, such as square, triangular, disks, and rings, have been investigated [23]. A single patch resonator typically supports TM_mn0_ modes. The feeding mechanism determines the types of modes that can be excited. Collinear feeding and orthogonal feeding have been used to design a square patch filter [24,25,26]. Colinear feeding excites only one of the fundamental modes (degenerate modes TM_100_ or TM_010_). However, orthogonal feed excites both modes, and a filter can be designed by adding a perturbation. Coupling between these modes can be achieved by adding an appropriate perturbation to the patch structure. Dual-mode operation allows a single resonator to achieve the equivalent performance of twofold coupled single-mode resonators, thereby reducing the size and complexity of the filter.

**Figure 1:**
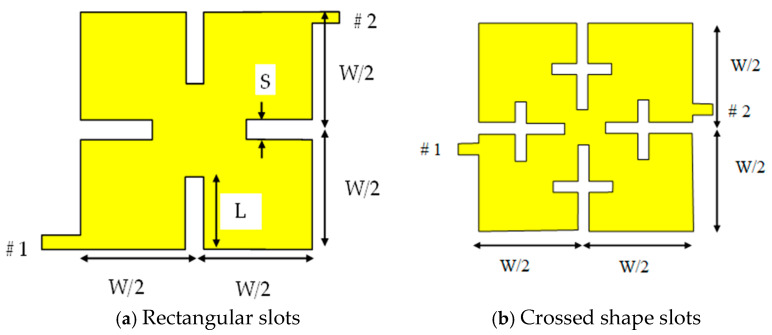
Topologies of nondegenerate dual-mode resonators.

A slotted patch resonator, as explained in [21,22], is used in this study, and is shown in Figure 1. Each of the proposed geometries can be employed as a dual-mode resonator. Figure 1a presents rectangular slots, while cross-shaped slots are shown in Figure 1b. The latter shape can provide better size reduction and a narrower bandwidth. Four symmetrical slots are etched into the square patch, positioned at half the patch width (W/2).

The slots perturb the current distribution across the patch, altering the effective resonant length and thereby controlling the filter’s frequency. Two asymmetrical feed lines are connected to opposite sides of the patch to excite multiple modes. Unlike conventional symmetric feeds, asymmetrical excitation generates both degenerate and higher-order modes within the same patch. This configuration enables multi-mode operation without requiring multiple coupled resonators. The feed lines are identical in length and width; their positions can also be varied to achieve impedance matching. The excited modes employed for this work are TM_100_, TM_010_, and TM_110_. TM_100_ and TM_010_ are degenerate modes that are excited at the same frequency; however, the TM_110_ mode is excited at a higher frequency. The slot length mainly defines the size reduction and the frequency difference between these modes. The slot lengths also determine the filter bandwidth.

By varying slot dimensions (length L, width S), the filter center frequency and passband bandwidth can be systematically adjusted. The resonance frequencies of the degenerate and higher-order modes are denoted as f_1_ and f_2_, respectively. The resonance frequencies f_1_ and f_2_ decrease as the slot’s length L increases. The resonance frequency f_1_ is affected by either vertical or horizontal slots; however, f_2_ is affected by both types of slots. Therefore, f_2_ decreases more rapidly than f_1_, allowing for the control of the filter bandwidth. The difference between f_2_ and f_1_ can approximate the filter bandwidth. The slot’s length L can mainly control this bandwidth. The filter center frequency can approximately be defined as the geometric mean of f_1_ and f_2_ ($\sqrt{f_{1}f_{2}}$).

The design concept can be applied at any band of the microwave spectrum. The degenerate dual-mode square patch resonator has a size of one-half waveguide wavelength (0.5λ_g_). However, the design of the suggested slotted square patch resonator can achieve one-quarter-waveguide wavelength (0.25λ). So, we expect to save an area as high as 75%. Therefore, the design of our proposed resonator began with an approximately square patch, with a length equal to one-quarter of the waveguide wavelength (0.25λ) at the lowest required tunable frequency. The selection of the substrate is flexible, depending on the requirements.

## 3. L-Band Reconfigurable Filter Design

**Figure 2:**
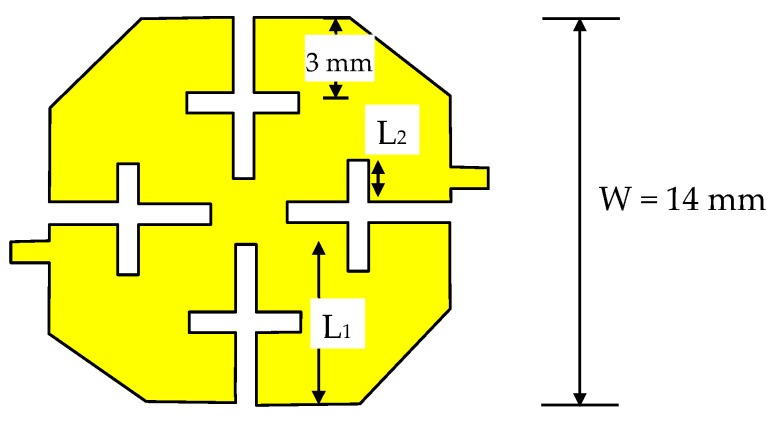
Dual-mode filter structure.

**Figure 3:**
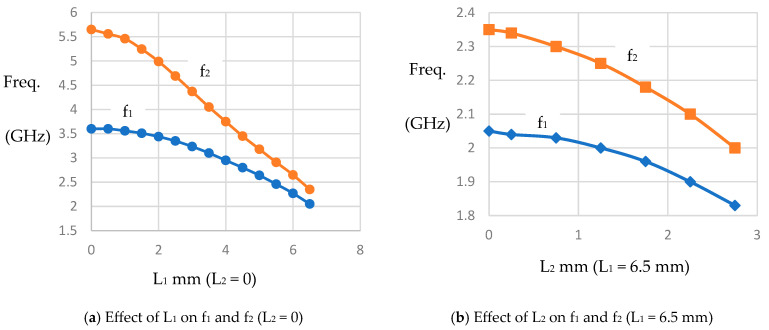
The effect of slot lengths L_1_ and L_2_ on the modes’ resonance frequencies f_1_ and f_2_.

**Figure 4:**
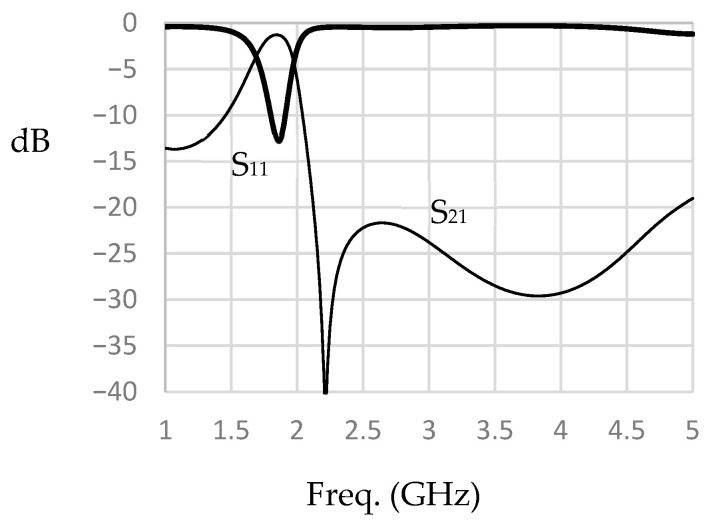
The characteristics of the filter before varactor loading.

The reconfigurable filter design under consideration starts with the design of a dual-mode filter at the end of the L-band. The description above is used in this design. RT/Duroid 6010.2 laminates with a 10.2 dielectric constant and 0.635 mm thickness are used to reduce the filter size. The structure in Figure 1b is first analyzed, but when loaded with varactor diodes at the corners, the stop band range is reduced significantly. For this reason, the cut corner configuration shown in Figure 2 is used instead. The patch size recommended to start the design is approximately one-quarter wavelength at the highest frequency (2 GHz). Based on the proposed substrate, W is chosen to be approximately 14 mm. Two types of slots, L_1_ and L_2_, as shown in the figure, are used. The corner cuts and etched slots are made symmetrically. Due to geometrical reasons, the maximum values of L_1_ and L_2_ should not exceed W/2 and W/4, respectively. L_2_ is positioned at approximately the middle of L_1_. The variation in the resonance frequencies f_1_ and f_2_ with L_1_, as calculated by electromagnetic simulation under the condition L_2_ = 0, is shown in Figure 3a. As explained earlier, it is found that f_1_ and f_2_ decrease as L_1_ increases, and f_2_ decreases faster. The slot width has little effect on f_1_ and f_2_ and is chosen in this design to be 0.5 mm. The variation in f_1_ and f_2_ against L_2_ when L_1_ is fixed at 6.5 mm is shown in Figure 3b. It can be demonstrated that f_1_ and f_2_ decrease to 1.83 GHz and 2 GHz, respectively, when L_2_ is 2.75 mm. Therefore, we expect a filter with L_1_ and L_2_ of 6.5 mm and 2.75 mm, respectively, to have an approximately passband range of 1.83 GHz to 2 GHz. A bandpass filter is designed under these conditions, and the response is shown in Figure 4. The minimum insertion loss is found to be 1.3 dB at 1.84 GHz. The −10 dB bandwidth is observed to be between 1.78 GHz and 1.93 GHz, and the 3 dB passband range of S_21_ is remarked to be between 1.7 GHz and 1.97 GHz. By loading this filter with varactor diodes, it is expected that the frequency band will decrease as the varactor capacitance increases, or in other words, as the reverse voltage decreases.

### Varactor Diode Loading Effect

**Figure 5:**
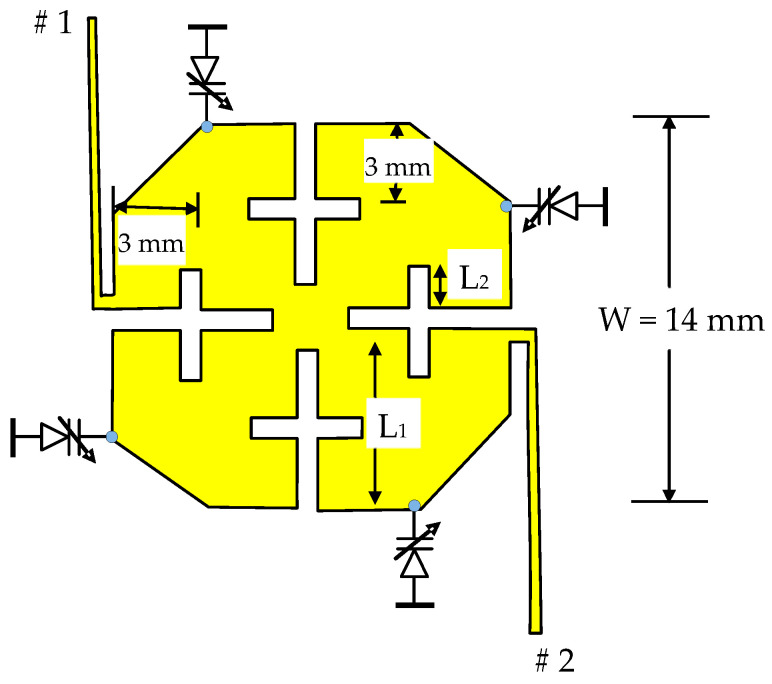
Varactor loading tunable dual-mode resonator.

**Figure 6:**
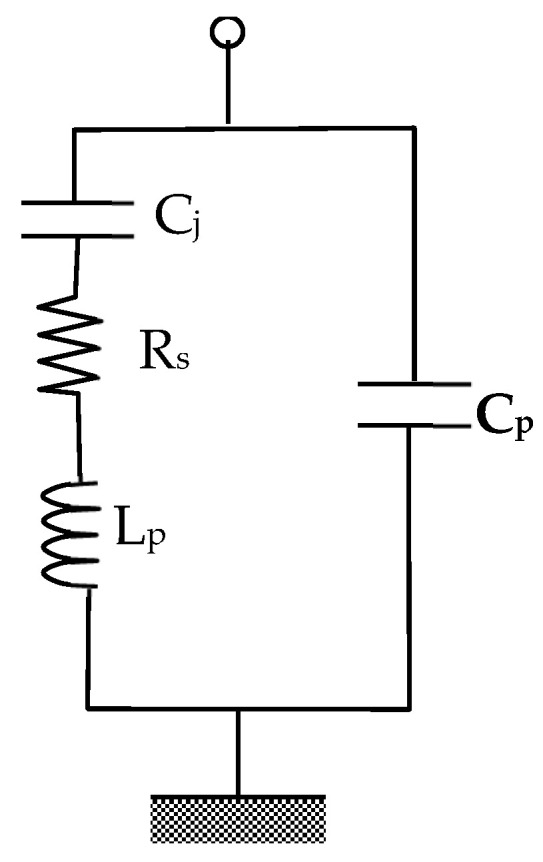
Varactor diode circuit.

Bandpass filter tunability can be performed by loading any of the nondegenerate dual-mode resonators shown in Figure 1 with four varactor diodes at the patch corners. The structure shown in Figure 5, with cut corners of 3 mm in size, is chosen to enhance the stopband characteristics. The feeding lines are typically used as quarter-wave impedance transformers for matching purposes. At the lower microwave frequency range, diodes in package form are generally used. In this case, the diodes’ parasitic parameters, L_P_ and C_P_, which affect the resonance frequencies f_1_ and f_2_, should be included. In this case, the varactor diode circuit model, as shown in Figure 6, is necessary for accurate simulation and design. L_p_ and C_P_ represent the package inductance and capacitance, while C_j_ and R_s_ represent the junction capacitance and the series resistance, respectively.

**Figure 7:**
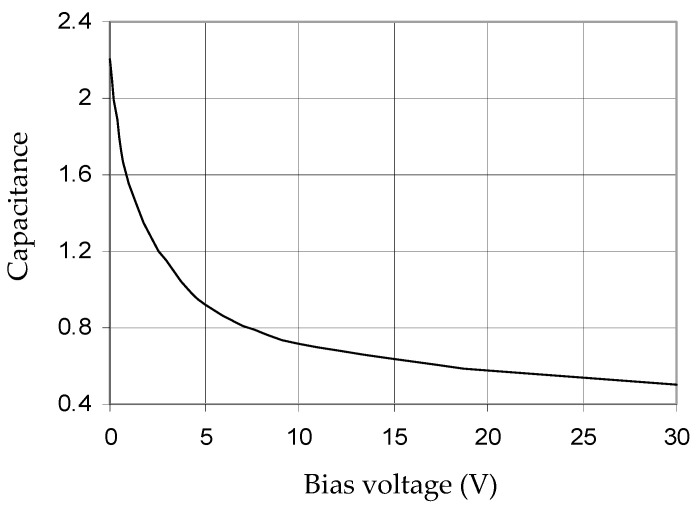
Typical capacitance of the SMTD3001 varactor (from Metelics Corp.) as a function of its reverse bias voltage [23].

In this paper, the silicon-based surface-mounted varactor diode SMTD3001 from Metelics Corp. (Sunnyvale, CA, USA) is used. The reverse bias capacitance characteristics of this diode are shown in Figure 7 [27]. The diode is suitable for operations up to 3 GHz in a temperature range of −65 °C to 150 °C. The diode junction capacitance varies from 2.25 pF to 0.5 pF for reverse bias voltage from 0 V to 30 V, respectively. Other varactor diodes, such as GVD30452 produced by Sprague—Goodman Electronics Inc. (Westbury, NY, USA), providing a higher capacitance ratio, can be used for a wide tunable band [28]. The diode parasitic parameters from the data sheet are L_p_ = 2 nH, and C_p_ = 0.1 pF. The junction capacitance located at the corners of the patch acts to increase the effective length of the excited modes, thereby controlling the resonator frequencies f_1_ and f_2_.

**Figure 8:**
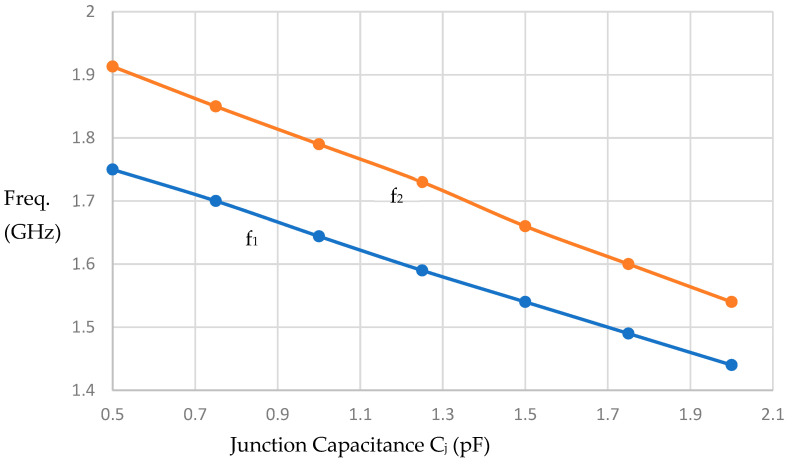
Variation in resonator resonance frequencies with the junction capacitance of the varactor diodes SMTD300 under reversed bias.

Loading the resonator in Figure 2 with four SMTD3001 reversed-bias varactor diodes at its corners will reduce the resonance frequencies f_1_ and f_2_. Figure 8 shows the effect of this loading when varactor junction capacitance C_j_ changes from 0.5 pF, corresponding to 30 V bias voltage to 2 pF corresponding to approximately 0.8 V. It can be shown that the resonance frequency f_1_ reduces from 1.75 GHz to 1.49 GHz when the junction capacitance varies from 0.5 pF to 2 pF and the resonance frequency f_2_ changes from 1.91 GHz to 1.6 GHz when junction capacitance varies from 0.5 pF to 2 pF.

## 4. Simulation and Experimental Results

**Figure 9:**
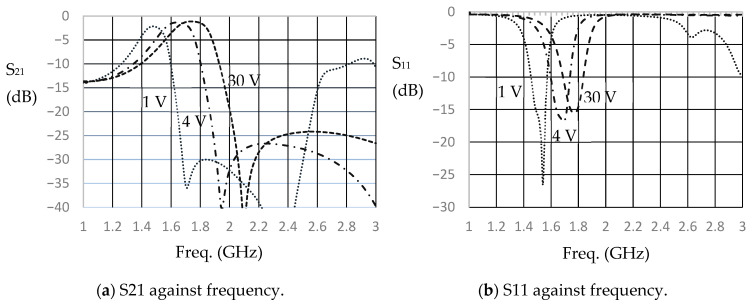
Simulation results of the reconfigurable filter.

The filter designed in the above section is loaded with four SMTD3001varactor diodes. The simulation is performed on the same substrate (RT/Duroid 6010.2) as mentioned in the previous section. The varactor model, as presented in Figure 6, is included. Three junction capacitance values of 0.5 pF, 1 pF, and 1.8 pF, corresponding to reverse bias voltages of approximately 30 V, 4 V, and 1 V, respectively, are used in the simulation. Figure 9a,b show the simulated S_21_ and S_11_, respectively. As shown in these figures, tunability is observed in the changes in the center frequency for the three cases, ranging from approximately 1.48 GHz (1.8 pF varactor capacitance) to 1.78 GHz (0.5 pF varactor capacitance). The relative bandwidth is noted to be approximately 10%. The filter with a 0.5 pF loading has a slightly higher bandwidth, as it is defined by the 3 dB bandwidth. But for saying −8 dB S_11_ bandwidth, it will be approximately 10%. The minimum insertion loss (IL) in the three-band increases as the varactor junction capacitance increases. The IL is found to be 1.14 dB at 1.78 GHz, 1.5 dB at 1.67 GHz, and 2.1 dB at 1.48 GHz. The increase in IL as the varactor capacitance increases is because of the varactor resistance.

**Figure 10:**
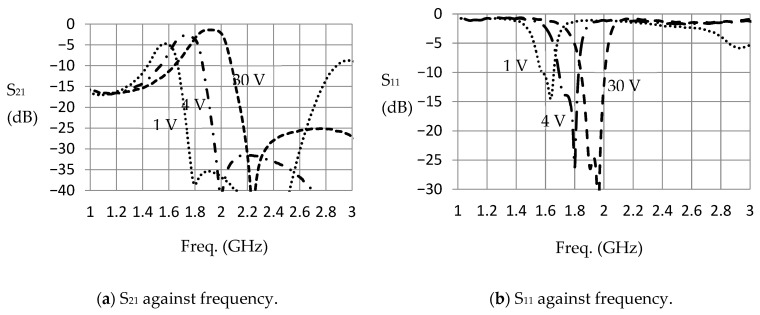
Measured results of the reconfigurable filter.

**Figure 11:**
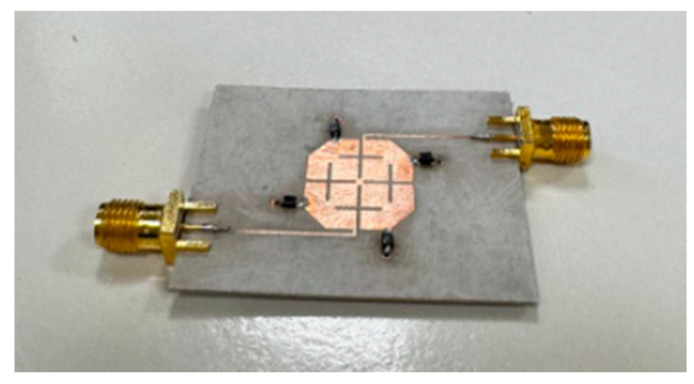
The photograph of the fabricated reconfigurable filter.

The filter is implemented on the same substrate. The S-parameter measurements are made using an Anritsu microwave Vector Network Analyzer (VNA 37369C, Anritsu Corporation, Atsugi, Japan). A bias Tee connected at one port is used to bias the varactor diodes. A quarter-wave transformer is applied at both ports to achieve matching. Figure 10a,b show the measured insertion loss (S_21_) and return loss (S_11_) of the fabricated filter, respectively. The measured IL in the three bands increases as the reverse bias voltage decreases, meaning the varactor junction capacitance increases. The IL is found to be 1.4 dB at 1.9 GHz, 2.7 dB at 1.73 GHz, and 4.7 dB at 1.57 GHz. A high in-band rejection level of approximately 30 dB is noted due to the transmission zero in the higher band. However, in the lower band, the rejection increases slowly to approximately 15 dB at 1 GHz. It is observed that a frequency shift of approximately 120 MHz occurs to the higher frequency. This may be attributed to several reasons, such as the accuracy of the substrate parameters, the varactor models, and the fabrication tolerance. The measured return loss is also found to be very good in the entire tuning range. The fabricated filter is shown in Figure 11. The overall performance of the presented filter is summarized in Table 1, along with a comparison to other tunable bandpass filters. The waveguide wavelength in this table is calculated at the lower band. In our case, the estimated frequency is 1.57 GHz. It can be observed that the proposed filter offers a significant size advantage, as well as wide tunable bandwidth, compared to others, especially those based on the dual-mode filter. Such filters find applications in multiband communication systems such as 5G and 6G wireless communications.

## 5. Conclusions

This paper presented the design and analysis of a compact tunable dual-mode microstrip patch bandpass filter. The filter combines symmetrical slots etched symmetrically on a square patch, asymmetrical feed lines, and varactor diodes. The symmetrical slots reduce the size of the patch resonator. At the same time, the asymmetrical feed lines enable excitation of both degenerate and higher-order modes, allowing a single square patch to function as a dual-mode resonator. The incorporation of varactor diodes as a loading mechanism enables continuous tuning of the filter center frequency through bias control of diode capacitance. A reconfigurable L-band filter is designed, simulated, and experimentally validated. High dielectric constant RT/Duroid 6010.2 laminates, with a dielectric constant of 10.2 and a thickness of 0.635 mm, are used to reduce the filter size. The surface-mount varactor diodes SMTD3001 are suitable for L-band applications.

The proposed design is suitable for various applications, including modern wireless communication, radar, and satellite systems.

## Figures and Tables

**Table 1:** Comparison of the proposed reconfigurable filter with some other filters in the literature.

Reference	Resonator Topology	Tuning Bandwidth BW @ f_0_ (MHz)	Fractional Bandwidth (%)	Fractional Tuning Range (%)	Insertion Loss (dB)	Size (λ_g_ × λ_g_)
[10]	Square Ring	220 @ 2330	NA	9.5	0.86	0.62
[11]	Rectangular Ring	220 @ 5600	5	4	0.5	0.09
[13]	Hairpin & Combline	175 @ 312.5	2.5	56	5.6–9.6	0.0069
[20]	Dual-Mode	232 @ 1200	10–15	18	2–3	0.0756
This Work	Dual-Mode	330 @1735	~10	19	1.4–4.7	0.055

## Data Availability

The data presented in this study are available upon request from the corresponding author.
